# Polyphosphate and Its Diverse Functions in Host Cells and Pathogens

**DOI:** 10.1371/journal.ppat.1003230

**Published:** 2013-05-02

**Authors:** Silvia N. J. Moreno, Roberto Docampo

**Affiliations:** 1 Center for Tropical and Emerging Global Diseases, University of Georgia, Athens, Georgia, United States of America; 2 Department of Cellular Biology, University of Georgia, Athens, Georgia, United States of America; University of Wisconsin Medical School, United States of America

Polyphosphate (polyP) is a linear polymer of a few to many hundreds of phosphate (P_i_) residues linked by high-energy phosphoanhydride bonds (Figure 1A). This ubiquitous polymer is found in bacteria, protists, and mammalian cells, and it was likely present prebiotically [1]. In bacteria, polyP accumulates in volutin or metachromatic granules, which are equivalent to acidocalcisomes [2]. In eukaryotic cells, polyP is present in different compartments, including the cytosol, nucleus, lysosomes, and mitochondria, but is preferentially accumulated in acidic vacuoles such as the yeast vacuole and acidocalcisomes [1], [3]. In these organelles, polyP, which is negatively charged, is in close association of inorganic (Ca^2+^, Mg^2+^, Zn^2+^, Fe^2+^, Na^+^, K^+^) and organic (basic amino acids, polyamines) cations. PolyP also combines with calcium and polyhydroxybutyrate forming channels in bacterial membranes, which make them competent for DNA entry; in mitochondria, as part of the mitochondrial permeability transition pore; and in the plasma membrane, as part of the potassium channel and the calcium pump (reviewed in [1], [4]). PolyP is arbitrarily divided into two forms: short-chain (from 3 to ∼300 P_i_) and long-chain (from 300 to ∼1000 P_i_) polyP, based on the method used for its extraction. For the detection of polyP, several methods have been described and a few examples are shown in Figure 1B–F.

**Figure 1:**
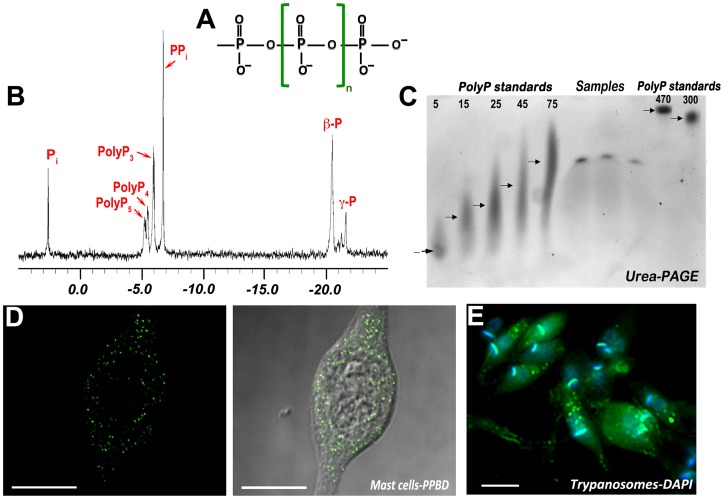
Methods used to detect polyP in cells. **(A)** Structure of polyP. **(B)** 303.6-MHz spectrum (^1^H decoupled) of a perchloric acid extract of isolated acidocalcisomes from epimastigotes of *T. cruzi*, showing peaks corresponding to P_i_; the α phosphates of pentapolyphosphate (polyP_5_), tetrapolyphosphate (polyP_4_), tripolyphosphate (polyP_3_); PP_i_; the β phosphates of tri-, tetra-, and pentapolyphosphate (β-P); and the γ phosphate (central) of pentapolyphosphate (γ-P). Reproduced with permission from reference [31], © the American Society for Biochemistry and Molecular Biology. **(C)** Urea-PAGE analysis of polyP from human platelets from three different donors. PolyP extracted from platelets was electrophoresed by 6% urea-PAGE. Chain lengths of standards are on the *left*. The lanes named “Samples" show the position of migration of samples from three different donors. Reproduced with permission from reference [22], © the American Society for Biochemistry and Molecular Biology. **(D)** Fluorescence analysis (*left*) and merge with bright field image (*right*) of the localization of polyP in mast cell granules (acidocalcisomes) using the recombinant polyP binding domain (PPBD) of *Escherichia coli* PPX linked with an Xpress epitope tag. Reproduced with permission from reference [23], © the American Society for Biochemistry and Molecular Biology. **(E)** DAPI staining of epimastigotes of *T. cruzi* showing the punctate staining of acidocalcisomes. Kinetoplast DNA is stained blue.

## Acidocalcisomes and PolyP

Acidocalcisomes were first described in trypanosomes and later found in Apicomplexan parasites, algae, slime molds, fungi, eggs of different origins, and human cells [3]. These organelles were originally described as acidic compartments storing high concentrations of calcium, and later work found that they are highly enriched in polyP [2]. As the description of acidocalcisomes progressed over the years, it was found that they are similar to the volutin or metachromic granules described in bacteria and are now considered to be the only organelles maintained over evolutionary time from bacteria to human cells [2]. Acidocalcisomes of some organisms are known to possess a number of pumps (H^+^-vacuolar pyrophosphatase, H^+^-vacuolar ATPase, Ca^2+^-ATPase), exchangers (Na^+^/H^+^; Ca^2+^/H^+^), channels (aquaporin, inositol 1,4,5-trisphosphate receptor (InsP_3_R)), and transporters that are necessary for their cation and water accumulation and release, as well as enzymes involved in the synthesis and degradation of pyrophosphate and polyP [3]. Recent studies have indicated that they belong to the group of lysosome-related organelles (reviewed in [5]). The recent discovery that they possess an InsP_3_R [6] suggests that they are also involved in Ca^2+^ signaling.

## Enzymes Involved in PolyP Metabolism

The concentration of polyP in cells is the result of the action of enzymes that catalyze the synthesis and degradation of this polymer—namely, the polyP kinases and the endo- (PPNs) and exopolyphosphatases (PPXs), respectively. Bacteria express one or two polyP kinases: PPK1, which catalyzes the reversible transfer of P_i_ from ATP to polyP and from polyP to ADP, and PPK2, which catalyzes the synthesis of polyP from GTP or ATP [7]. Bacteria also have PPXs but no PPNs [7]. Genes encoding eukaryotic PPNs [8] and PPXs [9] were initially reported in *Saccharomyces cerevisiae*. Recombinant PPXs from *Leishmania major* [10], *Trypanosoma cruzi* [11], and human cells (H-prune) [12] have also been characterized. A role for polyP in cancer has been proposed based on the role of the human PPX on tumor metastasis [12]. Another interesting finding in this regard is that polyP could activate mTOR kinase, a key step in the proliferation of mammary cancer cells [13]. A putative polyP kinase gene of bacterial origin (*DdPPK1*) was found in *Dictyostelium discoideum* [14] together with a second distinct polyP kinase (DdPPK2), which is apparently localized to the acidocalcisome [15]. DdPPK2 has a similar sequence and shares characteristics of actin-related proteins, which in turn are similar to muscle actins. Actin inhibitors such as phalloidin and DNase I also inhibit DdPPK2-mediated synthesis of polyP. Thus, this particular actin-related protein complex is an enzyme that can polymerize into an actin-like filament concurrent with its synthesis of a polyP chain in a fully reversible reaction [15]. Recent work in yeast identified the first eukaryotic enzyme involved in the synthesis and translocation of polyP to a vacuolar compartment: the vacuolar transporter chaperone 4, or VTC4 [16]. VTC4 is part of a complex of VTC proteins that are present in fungi, algae, trypanosomatids [17], and Apicomplexans [18], but is absent in mammalian cells. Another potential pathway for polyP synthesis in yeast is through the metabolism of inositol pyrophosphates (InsPP) [19], but it is not known whether this pathway is operative in other organisms. Yeast deficient in phosphoinositide phospholipase C (PI-PLC) are depleted of polyP and a pathway for polyP synthesis via InsPP (also known as diphosphoinositol polyphosphates) was postulated. Diphosphoinositol tetrakisphosphate (PP-IP_4_) is the precursor proposed for polyP synthesis [19]. The polyP synthesis pathway in mammalian cells is still unknown.

## Functions of PolyP

The function of polyP has been studied mainly in prokaryotes: as a P_i_ store, an energy source to replace ATP, in cation sequestration and storage, in cell membrane formation and function, in gene transcription control, in regulation of enzyme activities, in response to stress and stationary phase, and in the structure of channels and pumps (reviewed in [20]). Kornberg's group has also described the roles of polyP in the physiological adjustments of bacteria to growth, development, stress, and deprivation; its role in biofilm development, quorum sensing, and virulence; as well as in long-term survival and expression of virulence factors (reviewed in [1]). The functions of polyP in eukaryotic cells are not so well defined. Functions in apoptosis, enhancement of mitogenic activity of fibroblast growth factor, and in bone mineralization have been reviewed elsewhere [21]. Some critical discoveries about the function of polyP in mammalian cells have renewed interest in studying them in these cells. It was first demonstrated that polyP is stored in the dense granules of human platelets and in mast cell granules (acidocalcisomes) and released upon their activation [22], [23]. It was also shown that polyPs have a potent modulatory activity on blood coagulation [24] and inflammation [25]. Recent studies have demonstrated that polyP acts at four points in the blood-clotting cascade (reviewed by [26]). PolyP initiation of the contact pathway by activating Factor XII to Factor XIIa also leads to bradykinin formation by kallikrein-mediated high molecular weight kininogen cleavage [25]. Bradykinin is the ligand of kinin B2 receptor, which activates various intracellular signaling pathways that lead to inflammatory reactions (reviewed in [27]).

A function for polyP in adaptation to stress and osmoregulation has been assigned in less complex eukaryotic cells such as yeast, fungi, algae, and trypanosomes (reviewed in [28]). PolyP is particularly abundant in pathogenic fungi and trypanosomes. It accounts for nearly 40% of the total phosphate content of *S. cerevisiae* [9] and reaches levels >100 mM in P_i_ residues, assuming distribution across the entire volume of the cell, in trypanosomatids such as *Trypanosoma brucei*, *T. cruzi*, and *L. major* [2], and there are drastic changes in their levels upon osmotic stress [29].

## Role of PolyP in Pathogenesis

PolyP, which in bacteria is mainly of long-chain type (>300 and up to 1,000 P_i_ residues), has been reported to be important for virulence of different bacteria, such as *Salmonella* spp., *Shigella flexneri*, *Vibrio cholerae*, *Neisseria meningitides*, *Pseudomonas aeruginosa*, and *Mycobacterium tuberculosis*, but the mechanism involved is not known [30]. It has also been reported that conditions that decrease the levels of polyP in parasites such as *T. brucei*, *T. gondii*, or *L. major* (reviewed in [3]) reduce their pathogenicity. Whether this is due to osmotic fragility of the parasites as a result of changes in polyP levels that impact their ability to grow *in vivo*, making the immune response against them more successful, or to a role of polyP in modulating the immune response is not yet known.

## Concluding Remarks

The late Prof. Arthur Kornberg once stated [30]: “not only is polyP often absent from texts of biology and chemistry but, even when noticed, tends to be dismissed as a molecular fossil." Considering the wide distribution of this polymer and the diversity of functions that has been attributed to it, it is expected that future research will reveal new findings about this understudied compound. PolyP has been found in bacterial to human cells and has been reported to be important for virulence of different bacteria and a number of parasites, including those that cause toxoplasmosis, African trypanosomiasis, and leishmaniasis. Even more exciting are the findings about the role of polyP in cancer metastasis, blood coagulation, inflammation, and innate immunity. For example, a significant finding is that enzymes involved in polyP metabolism could be excellent targets for drug design not only against bacteria and parasites but also for regulation of important physiological and pathological processes such as coagulation, inflammation, innate immunity, and thrombosis.

## References

[1] Rao NN, Gomez-Garcia MR, Kornberg A (2009) Inorganic polyphosphate: essential for growth and survival. Annu Rev Biochem 78: 605–647. 19344251 10.1146/annurev.biochem.77.083007.093039

[2] Docampo R, de Souza W, Miranda K, Rohloff P, Moreno SN (2005) Acidocalcisomes - conserved from bacteria to man. Nat Rev Microbiol 3: 251–261. 15738951 10.1038/nrmicro1097

[3] Docampo R, Moreno SN (2011) Acidocalcisomes. Cell Calcium 50: 113–119. 21752464 10.1016/j.ceca.2011.05.012 PMC3156361

[4] Reusch RN (2012) Physiological importance of poly-(R)-3-hydroxybutyrates. Chem Biodivers 9: 2343–2366. 23161623 10.1002/cbdv.201200278

[5] Moreno SN, Docampo R (2009) The role of acidocalcisomes in parasitic protists. J Eukaryot Microbiol 56: 208–213. 19527347 10.1111/j.1550-7408.2009.00404.x PMC2802266

[6] Huang G, Bartlett PJ, Thomas AP, Moreno SNJ, Docampo R (2013) Acidocalcisomes of *Trypanosoma brucei* have an inositol 1,4,5-trisphosphate receptor that is required for growth and infectivity. Proc Natl Acad Sci U S A 110: 1887–1892. 23319604 10.1073/pnas.1216955110 PMC3562765

[7] Docampo R (2006) Acidocalcisomes and polyphosphate granules. In: Shively JM, editor. Microbiol Monograph: Inclusion in Prokaryotes. Berlin-Heidelburg: Springer-Verlag. pp. 53–70.

[8] Sethuraman A, Rao NN, Kornberg A (2001) The endopolyphosphatase gene: essential in *Saccharomyces cerevisiae* . Proc Natl Acad Sci U S A 98: 8542–8547. 11447286 10.1073/pnas.151269398 PMC37472

[9] Wurst H, Shiba T, Kornberg A (1995) The gene for a major exopolyphosphatase of *Saccharomyces cerevisiae* . J Bacteriol 177: 898–906. 7860598 10.1128/jb.177.4.898-906.1995 PMC176681

[10] Rodrigues CO, Ruiz FA, Vieira M, Hill JE, Docampo R (2002) An acidocalcisomal exopolyphosphatase from *Leishmania major* with high affinity for short chain polyphosphate. J Biol Chem 277: 50899–50906. 12393865 10.1074/jbc.M208940200

[11] Fang J, Ruiz FA, Docampo M, Luo S, Rodrigues JC, et al (2007) Overexpression of a Zn^2+^-sensitive soluble exopolyphosphatase from *Trypanosoma cruzi* depletes polyphosphate and affects osmoregulation. J Biol Chem 282: 32501–32510. 17827150 10.1074/jbc.M704841200

[12] Tammenkoski M, Koivula K, Cusanelli E, Zollo M, Steegborn C, et al (2008) Human metastasis regulator protein H-prune is a short-chain exopolyphosphatase. Biochemistry 47: 9707–9713. 18700747 10.1021/bi8010847

[13] Wang L, Fraley CD, Faridi J, Kornberg A, Roth RA (2003) Inorganic polyphosphate stimulates mammalian TOR, a kinase involved in the proliferation of mammary cancer cells. Proc Natl Acad Sci U S A 100: 11249–11254. 12970465 10.1073/pnas.1534805100 PMC208743

[14] Zhang H, Gomez-Garcia MR, Shi X, Rao NN, Kornberg A (2007) Polyphosphate kinase 1, a conserved bacterial enzyme, in a eukaryote, *Dictyostelium discoideum*, with a role in cytokinesis. Proc Natl Acad Sci U S A 104: 16486–16491. 17940044 10.1073/pnas.0706847104 PMC2034253

[15] Gomez-Garcia MR, Kornberg A (2004) Formation of an actin-like filament concurrent with the enzymatic synthesis of inorganic polyphosphate. Proc Natl Acad Sci U S A 101: 15876–15880. 15496465 10.1073/pnas.0406923101 PMC528760

[16] Hothorn M, Neumann H, Lenherr ED, Wehner M, Rybin V, et al (2009) Catalytic core of a membrane-associated eukaryotic polyphosphate polymerase. Science 324: 513–516. 19390046 10.1126/science.1168120

[17] Fang J, Rohloff P, Miranda K, Docampo R (2007) Ablation of a small transmembrane protein of *Trypanosoma brucei* (TbVTC1) involved in the synthesis of polyphosphate alters acidocalcisome biogenesis and function, and leads to a cytokinesis defect. Biochem J 407: 161–170. 17635107 10.1042/BJ20070612 PMC2049025

[18] Rooney PJ, Ayong L, Tobin CM, Moreno SN, Knoll LJ (2011) TgVTC2 is involved in polyphosphate accumulation in *Toxoplasma gondii* . Mol Biochem Parasitol 176: 121–126. 21195114 10.1016/j.molbiopara.2010.12.012 PMC3042031

[19] Lonetti A, Szijgyarto Z, Bosch D, Loss O, Azevedo C, et al (2011) Identification of an evolutionarily conserved family of inorganic polyphosphate endopolyphosphatases. J Biol Chem 286: 31966–31974. 21775424 10.1074/jbc.M111.266320 PMC3173201

[20] Kornberg A, Rao NN, Ault-Riche D (1999) Inorganic polyphosphate: a molecule of many functions. Annu Rev Biochem 68: 89–125. 10872445 10.1146/annurev.biochem.68.1.89

[21] Kulakovskaya TV, Vagabov VM, Kulaev IS (2012) Inorganic polyphosphate in industry, agriculture and medicine: Modern state and outlook. Process Biochem 47: 1–10.

[22] Ruiz FA, Lea CR, Oldfield E, Docampo R (2004) Human platelet dense granules contain polyphosphate and are similar to acidocalcisomes of bacteria and unicellular eukaryotes. J Biol Chem 279: 44250–44257. 15308650 10.1074/jbc.M406261200

[23] Moreno-Sanchez D, Hernandez-Ruiz L, Ruiz FA, Docampo R (2012) Polyphosphate Is a novel pro-inflammatory regulator of mast cells and is located in acidocalcisomes. J Biol Chem 287: 28435–28444. 22761438 10.1074/jbc.M112.385823 PMC3436523

[24] Smith SA, Mutch NJ, Baskar D, Rohloff P, Docampo R, et al (2006) Polyphosphate modulates blood coagulation and fibrinolysis. Proc Natl Acad Sci U S A 103: 903–908. 16410357 10.1073/pnas.0507195103 PMC1347979

[25] Müller F, Mutch NJ, Schenk WA, Smith SA, Esterl L, et al (2009) Platelet polyphosphates are proinflammatory and procoagulant mediators in vivo. Cell 139: 1143–1156. 20005807 10.1016/j.cell.2009.11.001 PMC2796262

[26] Morrissey JH, Choi SH, Smith SA (2012) Polyphosphate: an ancient molecule that links platelets, coagulation, and inflammation. Blood 119: 5972–5979. 22517894 10.1182/blood-2012-03-306605 PMC3383012

[27] Renne T (2012) The procoagulant and proinflammatory plasma contact system. Semin Immunopathol 34: 31–41. 21858560 10.1007/s00281-011-0288-2

[28] Docampo R, Ulrich P, Moreno SN (2010) Evolution of acidocalcisomes and their role in polyphosphate storage and osmoregulation in eukaryotic microbes. Philos Trans R Soc Lond B Biol Sci 365: 775–784. 20124344 10.1098/rstb.2009.0179 PMC2817225

[29] Ruiz FA, Rodrigues CO, Docampo R (2001) Rapid changes in polyphosphate content within acidocalcisomes in response to cell growth, differentiation, and environmental stress in *Trypanosoma cruzi* . J Biol Chem 276: 26114–26121. 11371561 10.1074/jbc.M102402200

[30] Kornberg A (2008) Abundant microbial inorganic polyphosphate, poly P kinase are underappreciated. Microbe Wash DC 3: 119–123.

[31] Moreno B, Urbina JA, Oldfield E, Bailey BN, Rodrigues CO, et al (2000) 31P NMR spectroscopy of *Trypanosoma brucei*, *Trypanosoma cruzi*, and *Leishmania major*. Evidence for high levels of condensed inorganic phosphates. J Biol Chem 275: 28356–28362. 10871617 10.1074/jbc.M003893200

